# Rejected sickness cash benefit claims after 180 days of sick leave in the Swedish rehabilitation chain: A nationwide register-based study

**DOI:** 10.1177/14034948241279949

**Published:** 2024-10-11

**Authors:** Ulrik Lidwall

**Affiliations:** 1 Department of Clinical Neuroscience, Karolinska Institutet, Sweden; 2 Department for Analysis, Swedish Social Insurance Agency, Sweden

**Keywords:** Sick leave, sickness insurance, claim, rejection, work ability

## Abstract

**Aim:**

Since a lack of financial security among vulnerable groups could further hamper health and well-being, this study scrutinises factors predicting rejected prolonged sickness cash benefit claims among people on compensated sick leave of more than 180 days with a rejection between days 181 and 365.

**Methods:**

All 246,872 claims for employed people on sick leave recorded in the Swedish official statistics register between January 2018 and June 2021 were analysed. Claim outcome was evaluated using logistic regression with odds ratios recalculated to relative risks (RR) with 95% confidence intervals (CI), mutually adjusted for sociodemographic, work and health-related factors.

**Results:**

Overall, 46,611 (19%) of the claims were rejected, with slightly lower rates among women (RR=0.97; 95% CI 0.95–0.99). Musculoskeletal diseases had the highest rejection rates (RR=1.84; 95% CI 1.75–1.94) followed by injuries (RR=1.57; 95% CI 1.50–1.64) and symptoms (RR=1.51; 95% CI 1.46–1.56). Mental disorders also had above-average rates (RR=1.14; 95% CI 1.09–1.19), whereas the lowest rates were found among pregnancy-related diagnoses (RR=0.13; 95% CI 0.12–0.14) and neoplasms (RR=0.18; 95% CI 0.18−0.18). Higher rates were found among immigrants (RR=1.37; 95% CI 1.34–1.40), those with only primary education (RR=1.09; 95% CI 1.06–1.12) and among blue-collar workers. The regional variation was substantial (RR range 0.41–1.72).

**Conclusions:**

**High rejection rates were found for complex diagnoses and diagnoses with presupposed work ability in physically lighter occupations and among groups with assumed precarious positions at the labour market. Systematic differences in rates were identified between geographic regions. More studies are warranted to conclude if the differences found could be justified by other factors.**

## Introduction

**Figure 1.**
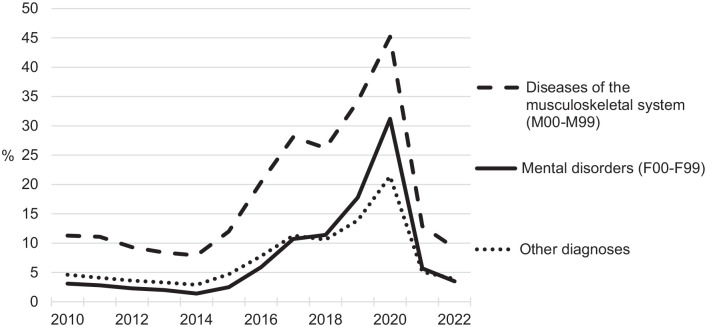
Proportion of rejected sickness cash benefit claims between days 181 and 365 in years 2010–2022 by diagnosis (ICD-10). Source: Swedish official statistics, Swedish Social Insurance Agency.

The cost of sick leave and disability in developed countries is high, and extensive policy actions have been taken to reduce the number of sickness and disability insurance recipients [1]. Still, there is a lack of firm evidence regarding the consequences of policy changes during the last decades in different countries – for instance, regarding the health and well-being for those who lose their social benefits [1]. In Sweden, a particular concern has been directed towards extensive sick leave due to mental disorders [2], and a policy response from the former right-wing government in order to reduce sick leave and increase labour supply was to introduce time limits in sickness insurance and stricter criteria for assessing work ability according to the ‘rehabilitation chain’ in 2008 [3–5]. In practice, this was a shift in responsibility from professionals representing organisations (e.g. case managers) to individuals experiencing reduced work ability [6]. In 2015, when sickness insurance expenditures had been rising again for some years, the left-wing government established a specific goal for the ‘sickness rate’ to be met by the end of 2020 [5,7]. Henceforth, the Swedish Social Insurance Agency (SSIA) applied a stricter assessment of work ability and insurance eligibility without contemporary changes in legislation [5,8], which subsequently decreased public trust in the sickness insurance system and the social insurance administration [5,9,10]. As a consequence of the stricter work ability assessments, the number of rejected sickness cash benefit claims rose substantially between 2015 and 2020, reaching its peak during the Covid-19 pandemic [11]. Rejection rates rose in general but were especially high for those still on sick leave after 180 days when their ability to work was evaluated in terms of available jobs throughout the entire labour market rather than in their current field of employment [4,5,10,12] (see Figure 1). Early in 2021, the left-wing government finally responded to the public distrust in sickness insurance with distinct legislative changes in sickness insurance, which increased eligibility, and rejection rates declined substantially thereafter [12].

Previous studies on rejected sickness or disability benefit claims for those with reduced work capacity due to impaired health have indicated that a fairly limited fraction actually return to gainful employment in contrast with policy expectations [3–5,13,14]. On the contrary, rejection of benefits claims have been reported to worsen mental health among already vulnerable groups in society [10,15–18]. The shift towards individual responsibility in the return-to-work process [6,19] could also be problematised in a health equity perspective, since impaired health and reduced work capacity is more common among the less educated in lower socio-economic strata [20–23]. Lower socio-economic status groups are also less well equipped with regard to health literacy, which is an important resource for maintaining and regaining health and work ability in complex societies and health-care systems [24]. In addition, the ability to navigate the social insurance system (i.e. social insurance literacy) is a more challenging task for those with less formal education and lower socio-economic status [25,26]. Hence, it could be expected that lower status groups in the labour market are at a higher risk of rejected sickness cash benefit claims. Up to 2019, the SSIA also requested so-called objective medical findings in the medical certificates to support the reduction in work ability [8]. Since objective medical findings are more often at hand for some medical conditions than others, higher rejection rates could also be expected among diseases with a more complex anamnesis and less objective findings, such as mental disorders and symptom diagnoses as reflected in official statistics [5,12,22,23]. In addition, it is expected that rejection rates could be higher for people with diseases of the musculoskeletal system as well as injuries because, according to the interpretation of the legislation at the time of the study, they could be considered to have work ability in less physically strenuous jobs [5,7,8,22,23].

There has been a long-lasting lack of transparency in the management of sickness insurance in Sweden since official rejection rates for sickness cash benefit were finally published six decades after the introduction of compulsory sickness insurance in 1955 [12]. In addition, surprisingly few studies have been published regarding rejected sickness cash benefits claims. Hence, the current study is a valuable contribution to the field.

### Swedish sickness insurance

The compulsory Swedish sickness insurance scheme potentially covers all Swedish or foreign citizens working in Sweden [11]. The prime requisite for being eligible for sickness cash benefit is that an individual has a disease or injury that reduces his/her ability to work by at least 25%. The first day of sick leave is a qualifying day without compensation, and sick pay is provided by the employer for the first 14 days. A medical certificate from a physician is required from day 8. If the reduction in work ability persists after 14 days, the employee receives sickness cash benefit from the national social insurance system. In 2008, specific time limits were introduced regarding the assessment of work ability, which gradually become stricter over time. For the first 90 days of sick leave, the impairment of the employee’s work capacity is assessed in relation to their regular work or other temporary work the employer can offer. After 90 days, the impairment of the employee’s work capacity is also assessed in relation to other jobs the employer can offer after redeployment. After 180 days of sick leave, the great divide occurs when you are only entitled to the benefit if you are unable to perform any job in the labour market. Several exceptions to the main rule apply, and these have been made more extensive since 2021 [11].

### Aim

The aim was to study individual factors predicting rejection of prolonged sickness cash benefit claims among people on compensated sick leave for at least 180 days with a rejected claim between day 181 and 365 in the sick leave period.

## Methods

Cases of sick leave compensated by compulsory Swedish sickness insurance were retrieved from the SToRE database, with data originating from administrative social insurance registers held by the SSIA. All spells exceeding 180 days with a claim and decision of prolonged sickness absence between 1 January 2018 and 30 June 2021 were included in the study. During this time, the number of rejected claims changed substantially (see Figure 1). The total number of cases was 246,872, with 162,388 for women and 84,484 for men.

### Measures

#### Dependent variable

Rejected sickness cash benefit claims after day 180 in the rehabilitation chain are defined as claims rejected between day 181 and 365 in the sick leave period – in short, the rejected benefit or rejection rate. This definition is identical to the definition applied in Swedish official social insurance statistics [12].

#### Predictors

For each case of sick leave, predictor variables were matched using the Swedish personal identity number. Predictor variables were sex, age, marital status, diagnosis, occupation, branch, employment sector, education, occupational status, origin of birth (Sweden or abroad), phase in time in relation to Covid-19 occurring during the study period and the subjects’ county of residence. Occupations were measured according to the Swedish version of ISCO-08 at the one-digit level with one exception: those in major group 5 (‘service, care and shop sales workers’) were analysed at the two-digit level, since group 5.3 (‘personal care workers’) essentially have different work characteristics compared to more commercial occupations within major group 5. Branches were measured according to the Swedish version of NACE v2 at the one-digit level. In addition to occupations and branches, information about subjects’ educational level (according to ISCED 1997), economic sector of their employer (private, state, regional or municipal authority) and occupational status (employed or not) were retrieved from Statistics Sweden. All other variables originate from the National Social Insurance Registers held by the SSIA. Diagnoses were analysed at the chapter level and were the latest primary sick leave diagnoses recorded on the medical certificate according to the Swedish version of ICD-10. For all other variables, data refer to the status at onset of the sick leave spell as recorded in the Swedish social insurance registers.

### Statistical analysis

All statistical analyses were performed using SAS EG v7.1. Logistic regression and corresponding 95% confidence intervals (CI) were used to analyse the odds of a rejected benefit claim after day 180. Since the outcome was rather common, odds ratios (OR) were recalculated to relative risks (RR) as suggested by Zhang and Yu [27]. Missing values for covariates constitute distinct categories in the analysis, but their results are not presented, since they lack meaningful interpretation. All the RRs presented were mutually adjusted for all other covariates included in the regression analysis.

## Results

Study population and outcome are presented in Table I. The majority of claimants were women, with a slightly lower rejection rate than men. Those aged 20–24 had higher rejection rates, and those aged 60–64 had lower rejection rates. Divorced people had somewhat higher rejection rates than married people.

**Table I:** Frequencies of covariates in the study population, outcome numbers and logistic regression results for rejected sickness cash benefits claims after day 180 in Sweden between January 2018 and June 2021.^a^.

Covariates	Study population		Rejected benefit claim		
	*n*	%	*n*	RR	95% CI
Sex					
Women	162,388	65.8	29,369	0.97	0.95–0.99
Men (reference)	84,484	34.2	17,242	1.00	–
Age (years)					
20–24	8450	3.4	1790	1.08	1.02–1.14
25–29	19,486	7.9	3579	1.01	0.97–1.05
30–34	23,371	9.5	4438	1.02	0.99–1.06
35–39	26,528	10.8	5007	1.01	0.98–1.03
40–44 (reference)	30,620	12.4	5831	1.00	–
45–49	33,183	13.4	6390	1.00	0.97–1.04
50–54	37,524	15.2	7267	0.98	0.95–1.01
55–59	36,430	14.8	7354	1.02	0.98–1.05
60–64	29,539	12.0	4673	0.85	0.82–0.89
⩾65	1741	0.7	282	1.00	0.89–1.11
Marital status					
Married (reference)	107,463	43.5	19,883	1.00	–
Unmarried	89,681	36.3	16,996	1.01	0.99–1.03
Divorced	45,375	18.4	8951	1.02	1.00–1.05
Widow/widower	4353	1.8	781	0.99	0.92–1.05
Diagnosis chapter ICD-10 (reference is the unweighted mean across diagnosis categories 1.00)					
I A00–B99 Certain infectious and parasitic diseases	1183	0.5	197	0.95	0.91–0.99
II C00–D48 Neoplasms	19,015	7.7	572	0.18	0.18–0.18
III D50–D89 Diseases of the blood and blood-forming organs	500	0.2	78	0.95	0.84–1.06
IV E00–E90 Endocrine, nutritional and metabolic diseases	1792	0.7	324	1.18	1.16–1.19
V F00–F99 Mental and behavioural disorders	117,555	47.6	20,756	1.14	1.09–1.19
VI G00–G99 Diseases of the nervous system	8426	3.4	1170	0.88	0.86–0.90
VII H00–H59 Diseases of the eye and adnexa	799	0.3	147	1.11	1.05–1.18
VIII H60–H95 Diseases of the ear and mastoid process	947	0.4	213	1.42	1.37–1.46
IX I00–I99 Diseases of the circulatory system	8173	3.3	1117	0.83	0.81–0.85
X J00–J99 Diseases of the respiratory system	3415	1.4	548	0.93	0.92–0.93
XI K00–K93 Diseases of the digestive system	2812	1.1	384	0.80	0.79–0.81
XII L00–L99 Diseases of the skin and subcutaneous tissue	890	0.4	201	1.33	1.28–1.37
XIII M00–M99 Diseases of the musculoskeletal system and connective tissue	46,496	18.8	14,200	1.84	1.75–1.94
XIV N00–N99 Diseases of the genitourinary system	1165	0.5	112	0.60	0.55–0.66
XV O00–O99 Pregnancy, childbirth and the puerperium	3944	1.6	94	0.13	0.12–0.14
XVIII R00–R99 Symptoms, signs and abnormal clinical and laboratory findings, not elsewhere classified	5132	2.1	1262	1.51	1.46–1.56
XIX S00–T98 Injury and poisoning	14,411	5.8	3863	1.57	1.50–1.64
Other (chapter XVI, XVII, XX and XXI)	4102	1.7	532	0.76	0.76–0.76
Unknown diagnosis	6115	2.5	841	–	–
Occupation (ISCO-08) (reference is the unweighted mean across occupations 1.00)					
0 Armed forces	116	0.1	19	1.11	0.78–1.51
1 Managers	9866	4.0	1511	0.84	0.82–0.86
2 Professionals	56,327	22.8	8704	0.85	0.81–0.88
3 Technicians and associate professionals	25,872	10.5	3833	0.83	0.79–0.86
4 Clerical support workers	18,893	7.6	2884	0.80	0.77–0.83
5.1 Personal services workers	6507	2.6	1417	1.00	0.98–1.02
5.2 Sales workers	9995	4.1	1943	0.98	0.95–1.00
5.3 Personal care workers	43,846	17.8	9779	1.04	1.00–1.08
5.4 Protective services workers	1741	0.7	346	1.03	1.00–1.05
6 Skilled agricultural and fishery workers	2123	0.9	491	1.15	1.13–1.17
7 Craft and related trades workers	16,420	6.6	3914	1.07	1.02–1.10
8 Plant and machine operators and assemblers	14,331	5.8	3427	1.10	1.06–1.14
9 Elementary occupations	13,991	5.7	3285	1.09	1.04–1.12
Occupation unknown	26,844	10.9	5058	–	–
Branch (NACE Rev. 2) (reference is the unweighted mean across branches 1.00)					
Land management (A)	2183	0.9	507	0.96	0.92–0.99
Manufacturing (B, C, D, E)	23,813	9.6	4271	0.79	0.78–0.80
Construction (F)	13,813	5.6	3533	1.07	1.06–1.08
Trade (G)	22,212	9.0	4283	0.95	0.94–0.96
Transportation (H)	10,412	4.2	2691	1.21	1.20–1.23
Hotel, restaurant, entertainment (I, R)	8971	3.6	2072	1.06	1.05–1.07
Business services (J, K, L, M, N, S)	43,942	17.8	7985	0.95	0.94–0.95
Public administration (O)	16,862	6.8	2502	1.01	1.00–1.01
Education (P)	32,762	13.3	6173	1.08	1.07–1.08
Social services (Q)	57,673	23.4	11,729	1.08	1.07–1.08
Branch unknown	14,229	5.8	865	–	–
Employment sector					
Private (reference)	126,728	51.3	26,245	1.00	–
Municipality	66,381	26.9	13,528	0.96	0.93–0.99
Region	19,917	8.2	3166	0.81	0.77–0.85
State	20,832	8.4	3038	0.74	0.71–0.78
Employment sector unknown	13,014	5.3	634	–	–
Education					
Primary education	27,584	11.17	6118	1.09	1.06–1.12
Secondary education (reference)	120,516	48.82	24,163	1.00	–
Post-secondary education <2 years	34,893	14.13	6038	0.99	0.97–1.02
Post-secondary education ⩾2 years	62,252	25.22	9920	1.00	0.97–1.03
Education unknown	1627	0.66	372	–	–
Occupational status					
Employed (work income, reference)	229,791	93.1	45,168	1.00	–
Not employed (no work income)	17,081	6.9	1443	0.88	0.82–0.94
Country of birth					
Sweden (reference)	202,707	82.1	35,637	1.00	–
Abroad	44,165	17.9	10,974	1.37	1.34–1.40
Phase in time in relation to Covid-19					
Pre-pandemic January 2018–February 2020 (reference)	162,539	65.8	26,452	1.00	–
Pandemic March 2020–February 2021	69,618	28.2	18,803	1.76	1.74–1.79
Post-pandemic March 2021–June 2021	14,715	6.0	1356	0.57	0.54–0.60
County of residence (reference is the unweighted mean across counties 1.00)					
Stockholm	54,324	22.0	10,332	0.76	0.71–0.81
Uppsala	10,369	4.2	2266	0.89	0.83–0.93
Södermanland	7498	3.0	865	0.43	0.39–0.46
Östergötland	9364	3.8	1017	0.41	0.37–0.44
Jönköping	9132	3.7	1859	0.84	0.79–0.89
Kronoberg	5113	2.1	992	0.82	0.76–0.86
Kalmar	5818	2.4	782	0.52	0.48–0.56
Gotland	1417	0.6	261	0.75	0.68–0.81
Blekinge	3576	1.4	752	0.95	0.89–1.00
Skåne	28,246	11.4	4497	0.62	0.58–0.66
Halland	7587	3.1	1538	0.85	0.79–0.90
Västra Götaland	47,095	19.1	7517	0.63	0.58–0.67
Värmland	6799	2.8	1726	1.15	1.08–1.19
Örebro	7043	2.8	1684	1.11	1.04–1.15
Västmanland	6891	2.8	1277	0.76	0.71–0.81
Dalarna	7409	3.0	1772	1.09	1.03–1.14
Gävleborg	7490	3.0	1787	1.01	0.95–1.05
Västernorrland	6323	2.6	1672	1.22	1.15–1.25
Jämtland	2957	1.2	991	1,72	1.66–1.74
Västerbotten	7204	2.9	1571	0.92	0.86–0.97
Norrbotten	5086	2.1	1406	1.30	1.23–1.34
Residence unknown	131	0.0	47	–	–

a All presented RRs were mutually adjusted for all other covariates presented in the table. ORs recalculated to RRs according to Zhang and Yu [27].

CI: confidence interval; RR: relative risk; OR: odds ratio.

There were substantial differences in rejection rates between different diagnostic categories. Low rejection rates were present for neoplasms, pregnancy-related diagnoses, genitourinary diseases and circulatory diseases. High rejection rates were found for musculoskeletal diseases, injuries, symptoms, diseases of the ear and eye, skin diseases, endocrine diseases and mental disorders. All other disease categories had rejection rates around the average.

A blue- versus white-collar occupational pattern was evident, with lower rejection rates among white-collar occupations in group 1–4 and higher rejection rates among blue-collar occupations in groups 5.3–9. Another pattern was found between branches, with higher than average rejection rates for those working within construction, transportation, hotel/restaurant/entertainment, education and social services compared to the lower than average rejection rates among those working within land management, manufacturing, trade and business services. Those working in private companies had higher rejection rates than those in public employment.

As for education, those with solely primary education had higher rejection rates than those with secondary education or higher. Those who were unemployed had lower rejection rates than those who were employed. Another evident difference was the higher rejection rate for those born abroad compared to native Swedes.

The higher rejection rates initiated from 2015 and culminated during the pandemic, with 76% higher levels during 2020 compared to preceding years. They then fell to 43% lower than pre-pandemic levels from March 2021.

Finally, there were substantial differences in rejection rates between residential regions. Lower rejection rates were found in more densely populated southern and eastern Sweden in counties such as Östergötland, Södermanland, Kalmar, Skåne, Västra Götaland, Stockholm and Västmanland. Higher rejection rates were present in more sparsely populated northern and western Sweden in counties such as Jämtland, Norrbotten, Västernorrland, Dalarna, Värmland and Örebro.

## Discussion

### Main findings

The sex difference in sick leave is substantial [11]. Still, the difference in rejected claims after more than 180 days of sick leave between the sexes is marginal, as reflected in official statistics [12], and the present study reports a 3% lower rate for women when a number of other factors are accounted for. Those aged 20–24 have higher rejection rates, and those aged 60–64 have lower rejection rates, which might reflect a presupposed work ability on the broader labour market [2]. The slightly higher rejection rates among divorced people may indicate a socially disadvantageous situation and economic stress [2], which could hamper people’s ability to make a claim to which they are entitled [24–26].

There are substantial differences in rejection rates after more than 180 days of sick leave between different diagnostic categories in line with expectations. Low rejection rates were present for diagnoses with distinct objective findings such as neoplasms, pregnancy-related diagnoses and genitourinary diseases. High rejection rates were found for musculoskeletal diseases, injuries, symptoms, diseases of the ear and eye, skin diseases, endocrine diseases and mental disorders. For mental disorders and symptoms diagnoses, this could possibly be due to the presupposed lack of objective medical findings and/or a bigger challenge for physicians to record adequately how such diagnoses reduce work ability as requested by the SSIA [5,8,22,23,28]. For other physical diagnoses with high rejection rates, there may also be an assumption that some residual work ability exists – for example, in physically lighter occupations for those with musculoskeletal diseases (e.g. back pain) or impairments due to injury [5,7,8,22,23].

Furthermore, the blue- versus white-collar occupational pattern is clear, with lower rejection rates after more than 180 days of sick leave among white-collar occupations and higher rejection rates among blue-collar occupations [7,10,23–26]. Another pattern was found between branches, with high rejection rates for those working within branches such as construction, transportation, hotel/restaurant/entertainment, education and social services (i.e. health care, childcare, care of elderly and disabled people, etc.). These branches have more adverse work environments and more extensive sick leave compared to low rejection and sick leave rates among subjects working within business services, manufacturing, trade and land management [2,21]. The higher rejection rates in certain occupations and branches are probably not a reflection of higher work ability in other jobs within the labour market but rather may reflect lower social insurance literacy [24–26]. Hence, the basis for decisions made by the SSIA have been questioned [5,8,10,26]. Subsequently, in September 2022, further legislative changes were initiated in sickness insurance, and assessments by the SSIA of employed people’s work ability have to be made in relation to specific occupations [11].

Moreover, those working in private companies have higher rejection rates after more than 180 days of sick leave than those working in the public sector. This could potentially be due to a lower degree of trade union organisation among employees in the private sector compared to the public sector [29] and consequently less organised legal support in claiming sickness insurance benefits.

In line with expectations, people with solely primary education have higher rejection rates than those with secondary education or higher [7,9,22,24,26]. Another evident difference is the higher rejection rates for those born abroad compared to native Swedes, which have been reported previously [7].

The fact that those who lack employment have lower rejection rates for prolonged compensation could be considered illogical, since the work ability of the unemployed is assessed with regard to jobs throughout the entire labour market from the first day of sick leave. However, the employment status for those on extensive sick leave probably reflects their health status and the fact that they have lost their previous employment due to long-standing illness and an inability to work [30].

The rejection rates after more than 180 days of sick leave rose from 2015 when the SSIA started to use the discretionary scope within contemporary sickness insurance legislation to reduce compensated sick leave in accordance with the specified goal set for the sickness rate [5,7,8,10]. The rejection rates culminated during the pandemic, and after legislative changes in March 2021, rejection rates fell sharply [4,12].

Finally, there are substantial differences in rejection rates between residential regions, reflecting the regional organisation of sickness insurance within the SSIA during the study period. Lower rejection rates were found in the more densely populated southern and eastern Sweden where sick leave rates are comparably lower [11]. Higher rejection rates are present in the more sparsely populated northern and western Sweden where sick leave rates are comparably higher [11]. An interpretation of the regional differences in rejection rates is that the discretionary scope within the contemporary sickness insurance legislation has been used to reduce prevailing regional differences in sick leave rates [5,11].

To sum up, rejection rates after more than 180 days of sick leave are higher for medically multifaceted diagnoses where the connection between illness and the reduction in work ability is more complex, given the presumption, during most of the study period, that this assessment is directed towards any job in the entire labour market. Furthermore, rejection rates are higher among disadvantaged groups in the labour market such as immigrants, the less educated and blue-collar workers.

Since several of the factors associated with higher rejection rates often coincide – for instance, for less educated, foreign born, privately employed blue-collar workers in branches with adverse work environments and musculoskeletal diseases – the actual rejection rates were probably substantial for some groups during the study period. The results from the current study also indicate that the stricter assessment of work ability employed by the SSIA potentially had negative health and welfare effects for vulnerable groups in the labour market, as indicated in earlier studies [3–5,10,14,15,17,18].

### Methodological considerations

This study has several major advantages in terms of generalisation. First, the data are generally very reliable, although registration errors cannot be ruled out entirely. Second, administrative data are not hampered by low response rates, as is often the case with standard survey techniques, and few cases had to be omitted due to missing values. Third, as all claims during the three-and-half-year study period for prolonged sick leave compensation after 180 days of sick leave made in the compulsory Swedish sickness insurance scheme were included, the validity is evident not only for the Swedish setting but also for comparable contexts. A further strength of the study is that a number of relevant confounders were accounted for in the regression analysis (e.g. age, diagnosis and occupation). Nevertheless, the study does have limitations. As in all observational studies, the possible impact of residual confounding from other unmeasured or poorly measured covariates cannot be excluded. However, there is no single factor that has not been included in the analyses that is a likely candidate to explain the main findings by confounding. Still, the observational nature of the study inherently opens up the possibility that other potential predictors influenced the outcomes.

## Conclusions

High rejection rates were found for complex diagnoses, diagnoses with presupposed work ability in physically lighter occupations and among groups with precarious positions in the labour market, indicating adverse welfare effects among vulnerable groups in society. In addition, systematic unexplained differences in rejection rates were identified between geographic regions, which raises questions about equality in Swedish sickness insurance. Further studies are warranted to conclude if the differences between groups and regions found in the present study are justified or if they can be attributed to other factors – for instance, substantial differences in reduced work ability beyond what is reflected in medical diagnoses.
